# Sociodemographic Characteristics and Health Profile of the Elderly Seeking Health Care in Kampala, Uganda

**DOI:** 10.1155/2018/4147509

**Published:** 2018-05-16

**Authors:** Faith Nawagi, Martin Söderberg, Vanja Berggren, Patrik Midlöv, Aidah Ajambo, Noeline Nakasujja

**Affiliations:** ^1^Euclid University Global Health Institute, Washington, DC, USA; ^2^Faculty of Social Sciences, Child Rights Institute, Lund University, Lund, Sweden; ^3^Research Group Clinical Health Promotion, Department of Health Sciences, Faculty of Medicine, Lund University, Lund, Sweden; ^4^Center for Primary Health Care Research, Lund University, Lund, Sweden; ^5^Makerere University-Johns Hopkins Research Collaboration, PMTCT Program, Kampala, Uganda; ^6^Department of Psychiatry, Makerere University, College of Health Sciences, Kampala, Uganda

## Abstract

Aging entails health challenges globally, but pertinent data from low-income countries like Uganda remains scarce. A cross-sectional study was carried out at Mulago National Referral Hospital in Kampala, among 134 patients (38% men and 62% women) aged ≥60 years. Data was collected on sociodemographic characteristics, medical disorders, cognitive function, hearing handicap, and functional status, that is, Basic Activities of Daily Living (BADL) and Instrumental Activities of Daily Living (IADL). The participants had high independency in BADL (89%) and IADL (75%). The most common medical conditions were bone/joint pain (35%), hypertension (24%), and visual problems (20%). More women (54%) than men (37%) reported bone and joint pain. The majority (80%) of the participants did not report any hearing handicap, and half (54%) did not have any cognitive impairment. Dependency in IADL was associated with advanced age, being female, and being financially dependent, and the risk of having a hearing handicap was higher among those above the median age (68 years). In adjusted models, the effects remained similar although statistical significance was only achieved for advanced age versus dependency in IADL (RR: 2.38, 95% CI: 1.12–5.08) and hearing handicap (RR: 2.67, 95% CI: 1.17–6.12). Thus, socioeconomic status and gender are relevant aspects when attempting to understand the health profile of the elderly in Kampala, Uganda.

## 1. Introduction

 Aging is a global population characteristic of the 21st century following improved health care systems and increased survival [1–3]. However, its wider implications in communities are one of the great challenges of this century. The elderly (aged ≥60 years) contribute 11% to the global population and this number is expected to increase to 20% by 2050 [4]. There has been a steady increase in the number of the elderly from 205 million in 1950 to 810 million in 2012, and this figure is expected to increase to two billion by 2050 [4, 5]. Sub-Saharan Africa contributes 5% (43 million) of the global elderly population and this percentage is expected to increase even higher by 2030 [5, 6]. In particular, urban areas are of special interest as the elderly population is growing faster here than in rural areas [3].

In Uganda, the elderly constitute 2.7% of the entire population [5, 6] and life expectancy has increased from 50 to 63 years over the past ten years [7]. The elderly constitute the poorest members of the society in Uganda with 64% living below the poverty line, with lack of access to regular income and little to no benefit from social security services [8]. Many live in poor housing coupled with poor nutrition and a high risk of suffering from various chronic conditions [6, 9–12]. Traditionally, health care has been hard to access due to poor health service delivery in public facilities, various costly private for-profit hospital settings, and a lack of specialized services for the elderly [13, 14]. These hindrances conform to the Andersen health care utilization model that elaborates the effect of predisposing factors such as race, health beliefs, enabling factors such as health financing, and need which refers to the personal and actual need of care [11]. In 2001, the Ugandan government abolished user fees in public hospitals after having instituted the policy for more than 4 years [15]. Nevertheless, health care-related costs still remain a major reason for postponing or abstaining from seeking care among the elderly [16–19]. Other deterrents may include poor accessibility of service points, poor availability of affordable drugs [19], perceived lack of skilled staff, and overall perceived substandard quality of the care offered [19, 20].

Four percent of the Ugandan 35-million population reside in Kampala, with a difference in urban rural demographic factors [21, 22], risk of injuries [23] and self-harm [24], and prevalence of disease, for example, diabetes mellitus [25]. Thus, studies performed on the Ugandan population as a whole may not necessarily be generalizable. Research performed on elderly residents of Kampala have focused mainly on HIV/AIDS, antiretroviral treatment, and caregiver roles [26–28], whereas the scientific literature documenting the overall health and quality of life among the elderly remains scanty.

The Ugandan government has put an effort to address the health needs of the elderly through the creation of a policy for elderly care, which led to the piloting of the rural based Social Assistance Grants for Empowerment (SAGE) program in 15 districts from 2010 to 2015 and an extension to 40 more districts out of 112 districts in Uganda by 2020 [11]. Under this scheme, initially registering any individuals over 65 years and now focusing on the oldest 100 persons in each district, a monthly stipend of 25000 Ugandan shillings (about 8 USD) is given after every 2 months to each elderly enrolled in the program [11]. However, this hardly meets the health and financial costs of these individuals.

With little scientific attention being paid to the elderly's health in Uganda's urban setting, this project aimed to assess the sociodemographic characteristics and health profiles of the elderly visiting Mulago, the national referral hospital of Uganda. A secondary aim was to compare the study population with the general Ugandan population of the elderly and national household statistics.

## 2. Materials and Methods

### 2.1. Study Setting

There are 155 hospitals in Uganda, of which 139 are general hospitals, 14 are regional referral hospitals, and two are national referral hospitals. One of the national referral hospitals (Butabika, Kampala) specializes in mental health services and mental health training, whereas the other (Mulago National Referral Hospital, MNRH) is the main health care provider in other areas of medicine. During the fiscal year 2014/2015, more than 800,000 outpatient visits and 750,000 inpatient care cases were recorded at MNRH. A diabetes and a hypertension clinic each operate at the hospital once a week. The Assessment Center at the MNRH Hospital is the first contact for both referred and nonreferred adult patients. It gets a high patient turnover with a minimum of 250 patients per day and is open Monday to Saturday from 8 a.m. to 5 p.m. but closed on Sundays and public holidays. The patients are triaged by clinicians and those with complicated conditions get referred for admission in the respective wards and emergency units, whereas outpatient cases are treated within the premises.

### 2.2. Study Design and Recruitment of Participants

This was a cross-sectional study conducted at the MNRH Assessment Center. Interviews were conducted in one of the clinical clerking rooms. Potential participants were identified upon the registration desk. Participants were also recruited from the medical outpatient clinics, that is, the hypertension and diabetes clinics, which were temporarily located at Kiruddu Health Centre due to reconstruction at the MNRH. The elderly who were critically ill, suffered from severe pain, were very lethargic, or otherwise had difficulties communicating were excluded from the study. Potential participants were briefed about the study, assessed for eligibility, and then enrolled if they fulfilled the selection criteria: aged ≥60 years, permanent residents of Kampala, and providing written informed consent. Sample size estimation was established using the Kish Leslie formula based on the 2015 world aging report that estimated 9.3% of the population in Africa to be elderly with a rapid growth in urban areas [2]. We assumed a statistical power of 80% and level of significance of 0.05 to obtain a sample size of 134 participants.

### 2.3. Data Collection

Data collection was performed by 10 research assistants (3rd-year medical students). These were trained by the principal investigator (FN) and a medical doctor (AA) on the standardized tools for elderly health care assessment used internationally to establish elderly health care needs and concerns. The functionality of the elderly was assessed using the Katz Index of Independence in Basic Activities of Daily Living (BADL) [29]. This instrument ranks performance in the 6 functions of bathing, dressing, toileting, transferring (moving to places), continence, and feeding. Participants were scored on yes/no responses, scoring 1 or 0 in each of the six functions. A score of 5-6 indicated full function, 3-4 moderate impairment, and 2–0 severe functional impairment. In addition to the IADL to assess functionality, the self-rated version of Lawton Instrumental Activities of Daily living scale (IADL) [30] was also used to measure functionality based on eight domains of activities: ability to use telephone, shopping ability, food preparation, housekeeping, laundry, transport/movement, taking medication, and ability to handle finances. Women were scored on all eight domains. Because some roles are gender-stereotyped, the areas of food preparation, housekeeping, and laundering were excluded for men. Therefore, men were assessed on five domains only.

The screening version of the Hearing Handicap Inventory for the Elderly (HHIE) [31] was used. This has ten questions concerning whether a hearing problem affected particular aspects of daily living. “Yes” was given a score of 4, “sometimes” a score of 2, and “no” a score of 0. Based on the standard categorization for the tool, scores were categorized such that 0–8 meant no self-perceived handicap, 10–22 mild to moderate perceived handicap, and 24–40 significant handicap.

The Mini-Mental State Examination (MMSE) [32] was used to assess the degree of cognitive impairment. The scores were interpreted as normal (25–30), mild impairment (20–25), moderate impairment (10–20), and severe impairment (0–10).

In addition, a questionnaire on social demographics of the participants was developed by the study team. The questions focused on age, gender, living arrangements, and income. Information on most pressing diseases and disorders and how much medical attention they had received was collected, in addition to whether this had been properly treated. Enquiry was also made into other health concerns.

Each research assistant was given a set of instruments to assess 15 elderly patients over a period of three weeks. The tools were interviewer-administered and handed over daily to the principal investigator who would do quality checks for completeness, give identifier numbers, and enter the information in the study database in IBM SPSS Statistics 23.0

### 2.4. Data Analysis

The potential associations between sociodemographic factors and outcome measures were evaluated using generalized linear models (GLM) with a Poisson distribution and a log-linear link, thereby estimating relative risks (RRs) with 95% confidence intervals (CIs). This analysis was performed for each sociodemographic characteristic in bivariate models, as well as including all sociodemographic characteristics (age, gender, household state, and financial status) in multivariate models in an effort to determine the effect sizes for each variable when taking the other factors into account. All outcome variables were analyzed in a dichotomized form such that any level of outcome was compared to absence of the outcome, for example, any cognitive impairment versus no cognitive impairment. All data was analyzed using IBM SPSS Statistics 23.0.

### 2.5. Ethical Clearance

The study was performed according to the guidelines set up in the Declaration of Helsinki. Ethical approval was obtained from the Institutional Review Board of Mulago Teaching and Referral Hospital (approval number: MREC; 987). All participants provided written informed individual consent with a signature or thumb print. Privacy and confidentiality were ensured during data collection through utilization of individual questionnaire administration and use of study identification numbers in place of participants' names.

## 3. Results

A 96% response rate was achieved through enrolling 134 participants (51 men and 83 women) out of the 140 elderly individuals who were contacted for participation.

### 3.1. Sociodemographic Characteristics

The majority of participants owned their own place of living, and half reported work as their main source of income as shown in Table 1. The women were younger, lived in smaller households, were more likely to live in a house owned by relatives, and were less likely have their main income from work as shown in Table 1. In comparison with national data, the male-to-female ratio was lower in the study population in all age groups (60–64 years: 79 versus 49; 65–69 years: 83 versus 72; 70–74 years: 71 versus 64; 75+ years: 72 versus 67). The age distribution differed from national data and people owning their own houses were overrepresented compared to national data for urban areas. The average household size was higher (72.8%) in the present study than in official statistics for urban Uganda. When comparing with data concerning people in the work force in Uganda as a whole, study participants aged 60–64 years as well as those aged 65+ years were less likely to work than their age peers (62% versus 85% and 44% versus 66%, resp.) [7, 33].

### 3.2. Disease Conditions Suffered by the Elderly

The most commonly reported age-related medical condition was bone/joint pain (*n* = 47, 35%) followed by hypertension (*n* = 32, 24%). Almost three-quarters (*n* = 96, 72%) claimed to be treating their disease appropriately as advised by the medical practitioners. Proper treatment was most common among those with diabetes (*n* = 4, 100%), with decreasing numbers for hypertension (*n* = 28, 88%), visual problems (*n* = 21, 78%), bone/joint pain (*n* = 32, 68%), urogenital problems (*n* = 2, 68%), and ulcers (*n* = 4, 44%). Almost half (*n* = 64, 48%) of the participants reported some form of pain. Women were more likely than men to report pain (*n* = 45, 54% versus *n* = 19, 37%).

### 3.3. Functional Status

The majority of the study population was independent in BADL and IADL and had no hearing handicap (80%) or cognitive impairment (54%) as shown in Table 2. There were no significant associations between the sociodemographic characteristics and dependency in BADL in either bivariate or multivariate models as shown in Table 3. Female gender, increasing age, and financial dependency were statistically associated with dependency in IADL at bivariate analysis as shown in Table 3, although only the effect of age remained statistically significant in multivariable analysis. Increasing age was a risk factor for hearing handicap, both in bivariate and in multivariate analysis. However, there were no statistically significant associations between the sociodemographic variables and cognitive impairment.

## 4. Discussion

### 4.1. Sociodemographic Characteristics

The sociodemographic patterns of the participants in this study describe the elderly population seeking health care in urban Uganda. Socioeconomic status has been found to be associated with health care utilization in Uganda and other low-income countries [1, 34, 35]. Risk factors for noncommunicable diseases have been found to vary both inversely and positively across the socioeconomic status gradient among adults in Uganda [36]. On the other hand, direct costs such as medication and consulting fees [37, 38], or indirect costs such as travels to service points and loss of income for time spent on the care visit [39], can both delay and inhibit health care utilization. Hence, the overrepresentation of the elderly of high socioeconomic status in the sample indicates that health care-related expenses may still prevent the elderly with low economic margins from seeking health care when afflicted by illness or injury. It is also important to note that this study focused on the profile of the elderly seeking care, not necessarily the factors that hinder them from health care utilization. Nevertheless, factors like age and socioeconomic status also affect health care utilization as predisposing and enabling factors according to the Andersen health care utilization model [11]. These factors could also have played a major role in determining which persons were recruited in this study given that participants were recruited from a hospital setting.

Women were overrepresented among those seeking health care, which is in agreement with previous studies in Uganda [40] and other parts of Africa [41, 42]. The higher health care utilization of women in this study may be attributable to more health care needs compared with men, yet it may also reflect a higher propensity among older women than men to seek health care. In the present setting, both explanations are plausible. Women live longer than men, which implies longer at-risk time for developing diseases and chronic disabilities [38]. In a nationally representative sample of Ugandans, women constituted a majority of the elderly (57% women versus 43% men) who reported being ill during the previous month [43]. Among middle-aged and elderly persons in northern Uganda seeking health care, gender has been found to be associated with different disease patterns [44]. For instance, older women had more hospital admissions for cancer and malaria, whereas AIDS, inguinal hernia, tuberculosis, femur fracture, and liver disease were more common among older men. Many hospital admissions among Ugandan women relate to child delivery and gynecoobstetrical conditions [44], and elderly women may have suffered long-term health effects of frequent pregnancies.

Besides biological explanations, the larger health care needs found among elderly Ugandan women may reflect gender differences in social conditions, that is, in terms of strain, workload, or variations in risk of contagion associated with responsibilities for kinship care [38]. In the African context, gender differences as social determinants of illness may be exemplified by the lower nutritional status found among women in households where men had complete control over cash income [45]. Furthermore, in Uganda, prioritization of household earnings has been found to be a critical determinant of health care seeking behavior. Compared with rural Ugandan women, urban women have relatively higher levels of financial independency and are thus more likely to attend hospitals [46]. A low availability of substitute labor, combined with pivotal responsibilities in the household, may also incite some women to seek health care to larger extents than men [38]. At the same time, among both men and women, higher levels of education are significantly linked to a shift from government-funded health care towards the more expensive private medical services, which in turn may reflect perceptions of the quality of the alternatives. Importantly, in researches that include admissions to private health clinics in measurements of access to health care, the overrepresentation of women among health care seekers is much less significant [37].

### 4.2. Disease Conditions Suffered by the Elderly

Bone and joint pain was highly prevalent and often untreated in the study population. This corroborates previous findings from low-income countries suggesting that experiences of bone pain are common [47] but often undertreated [47]. Bone and joint pain may have a profound effect on a person's life and can restrict daily activities [48, 49]. Therefore, proper treatment of bone ill-health and other pain inducing conditions is vital. Not to be forgotten are the hormonal changes (low estrogen levels which impact bone formation) that occur in menopause given that the majority of the study participants were women [36]. Nevertheless, the majority of the elderly in low-income countries eat low-calcium diets with many being ignorant about the foods rich in bone building nutrients like calcium and vitamin D. Bone health is one of the least prioritized arms of medicine in Uganda; the required tests are expensive and modalities to establish bone strength and illness are virtually nonexistent. For instance, in Uganda, the DXA scan that assesses bone mineral density is only available at Makerere University-Johns Hopkins (MUJHU) Research Collaboration solely for research purposes.

Hypertension was the second most commonly reported age-related medical condition. This is in agreement with previous findings that hypertension is highly prevalent among the elderly in sub-Saharan Africa [50–52]. Both in Africa and elsewhere, poor nutrition and lifestyle issues have been linked to increased risks of hypertension [53]. The number of people whose hypertension was being treated sufficiently was higher than that found in other studies carried out in sub-Saharan Africa [54]. The prevalence of hypertension in this study might be exaggerated due to selection bias given that some participants were recruited at hypertension clinics. This may also contribute to the high number of people who reported sufficient treatment for their hypertension.

Participants were assessed for potential hearing handicap, which should be distinguished from hearing impairment. A hearing impairment may be present without being considered a handicap. Although the majority of the participants in this study did not report any hearing handicap, the risk of such handicap was significantly increased among those above the median age (68 years). Hearing handicap increases the risk of deterioration in the quality of life for both the handicapped person [55–57] and his/her significant other [56]. Hearing aids have been found to alleviate hearing disability [58], as well as improving quality of life [59]. Thus, there is a need for specialized elderly health care to provide screening for hearing deterioration in order to allow correction of the impairment in the early stages.

Almost half of the participants had some form of cognitive impairment. This may partly be explained by the low literacy levels among Ugandan adults, especially among women [60], as literacy levels have been found to have a high correlation with MMSE scores [61]. Another potential explanation is the selection of participants, that is, people seeking health care. In previous studies, increasing age has been found to be associated with increased risk of cognitive impairment [62, 63], but there was no such association found in this study. This could partly be explained by the fact that half of the participants were still young elderly (i.e., under 68 years of age). Nevertheless, despite the limitations regarding assessment of cognitive impairment among study participants, this is—to the best of our knowledge—one of the few available data regarding cognitive function for the urban residing elderly in Uganda.

### 4.3. Functional Status

Even though cognitive decline was present in about half of the sample, the group still displayed high levels of independency in daily living. Affluent individuals may be better equipped to look after themselves and can thereby avoid complications of disease and thus maintain functional status. The majority of the participants in the present study were independent in BADL as well as IADL. As expected, independency was more common among those below the median age (68 years), though statistically significant only for IADL.

In univariate models, financial dependency was associated with dependency in IADL, a finding which is supported by previous studies in Uganda [64] and other low-income countries [65]. A weakness with all these studies is that they are cross-sectional and therefore provide no information on causality. It is reasonable to assume that a decreased functional status leads to dependency of others in several ways, of which financial dependency may be one. Nevertheless, arguments may also be made for a reversed causality, that is, that financial dependency leads to decreased functional status or an earlier onset of dependency in ADL. Longitudinal studies from developed countries have found a causal relationship between socioeconomic position and ADL [66]. However, measures of socioeconomic status may not necessarily be interpreted the same way in the African context [67]. Therefore, longitudinal studies set in sub-Saharan Africa are needed to elucidate the association between financial dependency and functional status among the elderly.

Women had lower independency (i.e., were more dependent) than men in IADL, which is in agreement with previous findings [68–70]. However, the opposite was found for BADL, a phenomenon which is not supported in existing literature regarding African populations [71, 72]. Thus, the women were more likely than men to be able to perform basic activities such as bathing and dressing, but less likely to be able to, for example, go shopping or prepare food. In the Ugandan setting, some of the activities included in the IADL are traditionally, regardless of age, carried out by the wives. Thus, it is reasonable to assume that failure to perform these will be less noticeable among men than among women. This could be a contributing reason for the gender differences found for IADL. With respect to BADL, one explanation for the higher independency among women could be the higher representation of women in this study. Part of the gender differences could possibly also be an effect of selection of patients in the study's hospital, in that men with independency in BADL may likely seek health care elsewhere.

### 4.4. Limitation of the Study

A few participants were recruited from special clinics for patients with diabetes and hypertension. Thus, there may be an overrepresentation of people with these conditions in the study population, which in turn could have contributed to the high number of individuals with hypertension. On the other hand, the number of individuals with diabetes was unexpectedly low.

Although we attempted to increase statistical power by dichotomizing the outcome variables, some of the analyses were based on rather small groups, which is reflected in the wide CIs. Thus, when interpreting the results, the effect size should be considered along with the statistical significance.

Since critically ill patients were omitted from the study, their health problems were not mapped. However, these patients were very few and their inclusion would most likely not have altered the study outcomes. Although every effort was made to ensure a calm testing environment, temporary health problems could have impacted the cognitive functioning and level of mental fatigue of respondents.

## 5. Conclusions

The major health concerns of the participants in this study included bone and joint pain, hypertension, visual problems, and moderate cognitive impairment. Since chronic bone and joint pain can have a severe negative impact on a person's quality of life, it is vital to ensure that proper treatment is available. This may be especially important among the elderly, as they in this regard constitute a particularly vulnerable population. Socioeconomic status impacts health care seeking behavior among the elderly in urban Uganda. This suggests that previous actions taken by the government, such as abolishing health care fees, have not—at least yet—had the intended effect. Mapping functional status and ill-health among the elderly seeking health care is important for medical services to calibrate their preparedness and capability to provide health care to this group. Uganda might benefit from a specialized arm of elderly health care. At the same time, it is imperative to address those elderly who need health care but for various reasons do not seek it. The seemingly complex and dynamic gender differences associated with both actual medical needs and relative utilization of health care should be paid further academic attention.

## Figures and Tables

**Table 1:** Sociodemographic characteristics of the 134 study participants and national data [7, 33].

	Study population			National data	
	Men	Women	Total	Total	Urban
*Age (years)*					
60–64; *n* (%)	15 (29)	31 (37)	46 (34)	31%	32%
65–69; *n* (%)	14 (27)	19 (23)	33 (25)	21%	20%
70–74; *n* (%)	7 (14)	11 (13)	18 (13)	19%	18%
75+; *n* (%)	15 (29)	22 (27)	37 (28)	30%	30%
*Owns his/her own living* ^*a*^					
Yes; *n* (%)	40 (78)	58 (70)	98 (73)	72.8%	42.8%
No; *n* (%)	11 (22)	25 (30)	36 (27)		
Rented; *n* (%)	6 (12)	6 (7)	12 (9)	21.4%	49.6%
With relatives; *n* (%)	5 (10)	19 (23)	24 (18)		
*Financially independent* ^*a*,*b*^					
Yes; *n* (%)	42 (84)	35 (44)	77 (60)		
Work; *n* (%)	38 (75)	29 (35)	67 (50)	95.8%	92.2%
Savings; *n* (%)	4 (8)	6 (7)	10 (8)	0.8%	1.4%
No; *n* (%)	8 (16)	44 (56)	52 (40)		
From relatives; *n* (%)	8 (16)	44 (54)	52 (39)	3.1%	6.1%
*Persons in the household* (mean)	6.3	5.5	5.8	4.7	4.0
*Monthly income *(1000 UGX) among those who work (median)	180	40	100		110

^a^National data are based on households, not individuals. ^b^Missing data for one man and three women.

**Table 2:** Functional status among the study participants.

	*n*	%
Katz Index of Independence in Activities of Daily Living		
No impairment	119	89
Moderate impairment	7	5
Severe impairment	8	6
Lawton-Brody Instrumental Activities of Daily Living Scale		
Independent	100	75
Dependent	34	25
Hearing Handicap Inventory for the Elderly-Screening Version (HHIE-S)		
No handicap	107	80
Mild to moderate handicap	21	16
Significant handicap	6	4
Mini-Mental State Examination (MMSE)		
None	72	54
Mild	32	24
Severe	30	22

**Table 3:** Independency in daily living and ill-health across sociodemographic variables among the 134 study participants.

	Dependency in BADL^a^	Dependency in IADL^b^	Hearing handicap	Cognitive decline
	*n* (%)	*n* (%)	*n* (%)	*n* (%)
*Gender*				
Men	8 (16)	6 (12)	8 (16)	19 (37)
Women	7 (8)	28 (34)	19 (23)	43 (52)
Women versus men^c^; RR (95% CI)	0.54 (0.20–1.48)	2.87 (1.19–6.93)	1.46 (0.64–3.33)	1.39 (0.81–2.39)
Women versus men^d^; RR (95% CI)	0.38 (0.12–1.26)	2.11 (0.81–5.50)	1.44 (0.59–3.53)	1.34 (0.73–2.46)
*Age*				
Below median (≤68 yrs)	6 (8)	13 (17)	9 (12)	32 (42)
Above median (>68 yrs)	9 (16)	21 (36)	18 (31)	30 (52)
Above versus below^c^; RR (95% CI)	1.97 (0.70–5.52)	2.12 (1.06–4.23)	2.62 (1.18–5.83)	1.23 (0.75–2.02)
Above versus below^d^; RR (95% CI)	1.99 (0.63–6.32)	2.38 (1.12–5.08)	2.67 (1.17–6.12)	1.22 (0.72–2.07)
*Accommodation*				
Rented/owned by relative	6 (17)	14 (39)	10 (28)	18 (50)
Owner	9 (9)	20 (20)	17 (17)	44 (45)
Owner versus renter^c^; RR (95% CI)	0.55 (0.20–1.55)	0.53 (0.27–1.04)	0.62 (0.29–1.36)	0.90 (0.52–1.55)
Owner versus renter^d^; RR (95% CI)	0.65 (0.20–2.07)	0.61 (0.29–1.31)	0.62 (0.27–1.45)	0.99 (0.55–1.80)
*Financially independent*				
No (depends on relatives)	7 (13)	21 (40)	15 (29)	29 (56)
Yes (work or savings)	7 (9)	10 (13)	12 (16)	30 (39)
Yes versus no^c^; RR (95% CI)	0.68 (0.24–1.93)	0.32 (0.15–0.68)	0.54 (0.25–1.15)	0.70 (0.42–1.16)
Yes versus no^d^; RR (95% CI)	0.57 (0.16–1.96)	0.54 (0.23–1.27)	0.80 (0.33–1.95)	0.80 (0.45–1.44)

^a^Basic activities of daily living. ^b^Instrumental activities of daily living. ^c^Bivariate analysis. ^d^Multivariate analysis.

## Data Availability

Datasets and tools developed for this study can be obtained from the corresponding author upon request.
